# Supplementary material for: Altered expression profile of glycolytic enzymes during testicular ischemia reperfusion injury is associated with the p53/TIGAR pathway: effect of fructose 1,6-diphosphate
Source: PeerJ. 2016 Jul 5;4:e2195. doi: 10.7717/peerj.2195 (PMC4941766; doi:10.7717/peerj.2195)
Supplement: Data S1 [file peerj-04-2195-s001.docx]

| **Taqman assays used for real-time PCR in this study** | | |
| --- | --- | --- |
| **Gene Name** | **Entrez Gene ID** | **Taqman Assay Reference #** |
| Hexokinase 1 | 25058 | Rn00562436_m1 |
| Glucose-6-phosphate isomerase | 292804 | Rn01475756_m1 |
| 6-Phosphofructokinase | 25741 | Custom made |
| Glyceraldehyde 3-phosphodehydroenase | 66020 | Rn01476455_m1 |
| Phosphoglycerate kinase | 316265 | Rn01511987_s1 |
| Lactate dehydrogebase C | 29634 | Rn00568562_m1 |
| PUMA | 317673 | Rn00597992_m1 |
| Survivin | 64041 | Rn00574012_m1 |
| Actin, beta | 81822 | Rn00667869_m1 |

**CT Values from real-time PCR experiments**

| **Contralateral** |  |  |
| --- | --- | --- |
|  |  |  |
|  | **HK1** | **b-Actin** |
| **Sham-C** | 16.09 | 15.90 |
|  | 16.05 | 15.88 |
|  | 15.87 | 15.95 |
|  | 17.18 | 16.91 |
|  | 15.88 | 15.98 |
|  | 16.51 | 16.67 |
| **Average** | **16.26** | **16.21** |
| **SD** | **0.51** | **1.10** |
|  |  |  |
| **tIRI-C** | 16.03 | 16.04 |
|  | 16.08 | 16.24 |
|  | 16.04 | 15.87 |
|  | 15.98 | 16.34 |
|  | 16.15 | 15.89 |
|  | 16.31 | 16.15 |
| **Average** | **16.10** | **16.09** |
| **SD** | **0.12** | **0.77** |
|  |  |  |
| **FBP-C** | 16.23 | 16.30 |
|  | 16.16 | 16.17 |
|  | 17.26 | 17.25 |
|  | 16.44 | 16.32 |
|  | 16.23 | 16.30 |
|  | 16.72 | 16.65 |
| **Average** | **16.51** | **16.50** |
| **SD** | **0.42** | **0.40** |
|  |  |  |
|  |  |  |
|  |  |  |
|  |  |  |
| **Ipsilateral** |  |  |
|  |  |  |
|  | **HK1** | **b-Actin** |
| **Sham-I** | 18.61 | 19.08 |
|  | 17.79 | 18.20 |
|  | 17.45 | 17.89 |
|  | 18.04 | 18.99 |
|  | 17.40 | 17.95 |
|  | 17.65 | 18.55 |
| **Average** | **17.83** | **18.44** |
| **SD** | **0.45** | **0.51** |
|  |  |  |
| **tIRI-I** | 17.67 | 17.81 |
|  | 18.05 | 17.65 |
|  | 18.79 | 19.02 |
|  | 17.39 | 17.43 |
|  | 18.63 | 18.58 |
|  | 17.81 | 18.13 |
| **Average** | **18.06** | **18.10** |
| **SD** | **0.55** | **0.60** |
|  |  |  |
| **FBP-I** | 17.94 | 18.32 |
|  | 17.99 | 18.47 |
|  | 17.10 | 18.25 |
|  | 18.84 | 19.39 |
|  | 18.22 | 18.39 |
|  | 17.82 | 18.29 |
| **Average** | **17.98** | **18.52** |
| **SD** | **0.57** | **0.43** |

| **Contralateral** |  |  |
| --- | --- | --- |
|  |  |  |
|  | **GPI** | **b-Actin** |
| **Sham-C** | 19.83 | 15.90 |
|  | 19.67 | 15.88 |
|  | 20.16 | 15.95 |
|  | 20.82 | 16.91 |
|  | 20.13 | 15.98 |
|  | 21.69 | 16.67 |
| **Average** | **20.38** | **16.21** |
| **SD** | **0.75** | **1.10** |
|  |  |  |
| **tIRI-C** | 19.99 | 16.04 |
|  | 20.87 | 16.24 |
|  | 20.05 | 15.87 |
|  | 20.07 | 16.34 |
|  | 19.86 | 15.89 |
|  | 19.64 | 16.15 |
| **Average** | **20.08** | **16.09** |
| **SD** | **0.42** | **0.77** |
|  |  |  |
| **FBP-C** | 20.53 | 16.30 |
|  | 20.71 | 16.17 |
|  | 20.90 | 17.25 |
|  | 20.89 | 16.32 |
|  | 20.73 | 16.30 |
|  | 20.22 | 16.65 |
| **Average** | **20.66** | **16.50** |
| **SD** | **0.26** | **0.40** |
|  |  |  |
|  |  |  |
|  |  |  |
|  |  |  |
| **Ipsilateral** |  |  |
|  |  |  |
|  | **GPI** | **b-Actin** |
| **Sham-I** | 20.08 | 19.08 |
|  | 20.45 | 18.20 |
|  | 20.46 | 17.89 |
|  | 20.20 | 18.99 |
|  | 20.37 | 17.95 |
|  | 21.45 | 18.55 |
| **Average** | **20.50** | **18.44** |
| **SD** | **0.49** | **0.51** |
|  |  |  |
| **tIRI-I** | 20.21 | 17.81 |
|  | 20.30 | 17.65 |
|  | 20.88 | 19.02 |
|  | 20.14 | 17.43 |
|  | 20.15 | 18.58 |
|  | 20.13 | 18.13 |
| **Average** | **20.30** | **18.10** |
| **SD** | **0.29** | **0.60** |
|  |  |  |
| **FBP-I** | 20.41 | 18.32 |
|  | 20.50 | 18.47 |
|  | 20.91 | 18.25 |
|  | 21.12 | 19.39 |
|  | 20.64 | 18.39 |
|  | 20.52 | 18.29 |
| **Average** | **20.68** | **18.52** |
| **SD** | **0.28** | **0.43** |

| **Contralateral** |  |  |
| --- | --- | --- |
|  |  |  |
|  | **PFK** | **b-Actin** |
| **Sham-C** | 20.97 | 15.90 |
|  | 20.50 | 15.88 |
|  | 19.90 | 15.95 |
|  | 21.36 | 16.91 |
|  | 20.26 | 15.98 |
|  | 21.17 | 16.67 |
| **Average** | **20.69** | **16.21** |
| **SD** | **0.56** | **1.10** |
|  |  |  |
| **tIRI-C** | 20.69 | 16.04 |
|  | 20.34 | 16.24 |
|  | 20.02 | 15.87 |
|  | 19.94 | 16.34 |
|  | 21.15 | 15.89 |
|  | 20.40 | 16.15 |
| **Average** | **20.42** | **16.09** |
| **SD** | **0.45** | **0.77** |
|  |  |  |
| **FBP-C** | 20.61 | 16.30 |
|  | 20.95 | 16.17 |
|  | 21.64 | 17.25 |
|  | 20.56 | 16.32 |
|  | 19.99 | 16.30 |
|  | 20.61 | 16.65 |
| **Average** | **20.73** | **16.50** |
| **SD** | **0.54** | **0.40** |
|  |  |  |
|  |  |  |
|  |  |  |
|  |  |  |
|  |  |  |
|  |  |  |
| **Ipsilateral** |  |  |
|  |  |  |
|  | **PFK** | **b-Actin** |
| **Sham-I** | 20.26 | 19.08 |
|  | 21.61 | 18.20 |
|  | 20.15 | 17.89 |
|  | 21.30 | 18.99 |
|  | 20.81 | 17.95 |
|  | 20.67 | 18.55 |
| **Average** | **20.80** | **18.44** |
| **SD** | **0.57** | **0.51** |
|  |  |  |
| **tIRI-I** | 20.86 | 17.81 |
|  | 20.93 | 17.65 |
|  | 21.60 | 19.02 |
|  | 20.23 | 17.43 |
|  | 21.92 | 18.58 |
|  | 20.89 | 18.13 |
| **Average** | **21.07** | **18.10** |
| **SD** | **0.60** | **0.60** |
|  |  |  |
| **FBP-I** | 21.05 | 18.32 |
|  | 20.86 | 18.47 |
|  | 21.13 | 18.25 |
|  | 21.91 | 19.39 |
|  | 20.73 | 18.39 |
|  | 20.41 | 18.29 |
| **Average** | **21.01** | **18.52** |
| **SD** | **0.51** | **0.43** |

| **Contralateral** |  |  |
| --- | --- | --- |
|  |  |  |
|  | **GAPDHS** | **b-Actin** |
| **Sham-C** | 15.70 | 15.90 |
|  | 15.72 | 15.88 |
|  | 15.54 | 15.95 |
|  | 16.55 | 16.91 |
|  | 15.71 | 15.98 |
|  | 16.30 | 16.67 |
| **Average** | **15.92** | **16.21** |
| **SD** | **0.41** | **1.10** |
|  |  |  |
| **tIRI-C** | 15.74 | 16.04 |
|  | 15.97 | 16.24 |
|  | 15.42 | 15.87 |
|  | 15.93 | 16.34 |
|  | 15.68 | 15.89 |
|  | 15.87 | 16.15 |
| **Average** | **15.77** | **16.09** |
| **SD** | **0.20** | **0.77** |
|  |  |  |
| **FBP-C** | 16.03 | 16.30 |
|  | 15.86 | 16.17 |
|  | 16.85 | 17.25 |
|  | 16.07 | 16.32 |
|  | 15.80 | 16.30 |
|  | 16.17 | 16.65 |
| **Average** | **16.13** | **16.50** |
| **SD** | **0.38** | **0.40** |
|  |  |  |
|  |  |  |
|  |  |  |
|  |  |  |
| **Ipsilateral** |  |  |
|  |  |  |
|  | **GAPDHS** | **b-Actin** |
| **Sham-I** | 15.83 | 19.08 |
|  | 16.11 | 18.20 |
|  | 17.27 | 17.89 |
|  | 16.99 | 18.99 |
|  | 17.43 | 17.95 |
|  | 17.33 | 18.55 |
| **Average** | **16.83** | **18.44** |
| **SD** | **0.68** | **0.51** |
|  |  |  |
| **tIRI-I** | 16.89 | 17.81 |
|  | 16.40 | 17.65 |
|  | 17.15 | 19.02 |
|  | 16.80 | 17.43 |
|  | 17.51 | 18.58 |
|  | 16.85 | 18.13 |
| **Average** | **16.93** | **18.10** |
| **SD** | **0.37** | **0.60** |
|  |  |  |
| **FBP-I** | 16.02 | 18.32 |
|  | 17.01 | 18.47 |
|  | 17.18 | 18.25 |
|  | 17.77 | 19.39 |
|  | 17.08 | 18.39 |
|  | 17.12 | 18.29 |
| **Average** | **17.03** | **18.52** |
| **SD** | **0.57** | **0.43** |

| **Contralateral** |  |  |
| --- | --- | --- |
|  |  |  |
|  | **PGK2** | **b-Actin** |
| **Sham-C** | 17.37 | 15.90 |
|  | 17.18 | 15.88 |
|  | 17.20 | 15.95 |
|  | 17.76 | 16.91 |
|  | 17.01 | 15.98 |
|  | 17.51 | 16.67 |
| **Average** | **17.34** | **16.21** |
| **SD** | **0.27** | **1.10** |
|  |  |  |
| **tIRI-C** | 17.20 | 16.04 |
|  | 17.41 | 16.24 |
|  | 17.12 | 15.87 |
|  | 17.24 | 16.34 |
|  | 17.52 | 15.89 |
|  | 17.51 | 16.15 |
| **Average** | **17.33** | **16.09** |
| **SD** | **0.17** | **0.77** |
|  |  |  |
| **FBP-C** | 17.04 | 16.30 |
|  | 17.45 | 16.17 |
|  | 17.73 | 17.25 |
|  | 17.97 | 16.32 |
|  | 17.51 | 16.30 |
|  | 17.25 | 16.65 |
| **Average** | **17.49** | **16.50** |
| **SD** | **0.33** | **0.40** |
|  |  |  |
|  |  |  |
|  |  |  |
|  |  |  |
| **Ipsilateral** |  |  |
|  |  |  |
|  | **PGK2** | **b-Actin** |
| **Sham-I** | 17.32 | 19.08 |
|  | 17.84 | 18.20 |
|  | 17.05 | 17.89 |
|  | 17.99 | 18.99 |
|  | 17.85 | 17.95 |
|  | 17.75 | 18.55 |
| **Average** | **17.63** | **18.44** |
| **SD** | **0.36** | **0.51** |
|  |  |  |
| **tIRI-I** | 17.06 | 17.81 |
|  | 17.07 | 17.65 |
|  | 17.69 | 19.02 |
|  | 17.01 | 17.43 |
|  | 17.24 | 18.58 |
|  | 17.31 | 18.13 |
| **Average** | **17.23** | **18.10** |
| **SD** | **0.25** | **0.60** |
|  |  |  |
| **FBP-I** | 17.80 | 18.32 |
|  | 17.52 | 18.47 |
|  | 17.24 | 18.25 |
|  | 17.65 | 19.39 |
|  | 17.31 | 18.39 |
|  | 17.28 | 18.29 |
| **Average** | **17.47** | **18.52** |
| **SD** | **0.23** | **0.43** |

| **Contralateral** |  |  |
| --- | --- | --- |
|  |  |  |
|  | **LDHC** | **b-Actin** |
| **Sham-C** | 16.52 | 15.90 |
|  | 16.18 | 15.88 |
|  | 16.39 | 15.95 |
|  | 17.24 | 16.91 |
|  | 16.39 | 15.98 |
|  | 17.04 | 16.67 |
| **Average** | **16.62** | **16.21** |
| **SD** | **0.42** | **1.10** |
|  |  |  |
| **tIRI-C** | 16.38 | 16.04 |
|  | 16.36 | 16.24 |
|  | 16.37 | 15.87 |
|  | 16.28 | 16.34 |
|  | 16.61 | 15.89 |
|  | 16.58 | 16.15 |
| **Average** | **16.43** | **16.09** |
| **SD** | **0.13** | **0.77** |
|  |  |  |
| **FBP-C** | 16.27 | 16.30 |
|  | 16.19 | 16.17 |
|  | 17.04 | 17.25 |
|  | 16.71 | 16.32 |
|  | 17.09 | 16.30 |
|  | 16.99 | 16.65 |
| **Average** | **16.72** | **16.50** |
| **SD** | **0.40** | **0.40** |
|  |  |  |
|  |  |  |
|  |  |  |
|  |  |  |
| **Ipsilateral** |  |  |
|  |  |  |
|  | **LDHC** | **b-Actin** |
| **Sham-I** | 16.74 | 19.08 |
|  | 17.17 | 18.20 |
|  | 17.72 | 17.89 |
|  | 17.62 | 18.99 |
|  | 16.84 | 17.95 |
|  | 17.50 | 18.55 |
| **Average** | **17.27** | **18.44** |
| **SD** | **0.41** | **0.51** |
|  |  |  |
| **tIRI-I** | 17.14 | 17.81 |
|  | 17.76 | 17.65 |
|  | 18.51 | 19.02 |
|  | 16.57 | 17.43 |
|  | 16.94 | 18.58 |
|  | 17.06 | 18.13 |
| **Average** | **17.33** | **18.10** |
| **SD** | **0.70** | **0.60** |
|  |  |  |
| **FBP-I** | 16.95 | 18.32 |
|  | 17.56 | 18.47 |
|  | 17.55 | 18.25 |
|  | 17.64 | 19.39 |
|  | 16.98 | 18.39 |
|  | 16.88 | 18.29 |
| **Average** | **17.26** | **18.52** |
| **SD** | **0.36** | **0.43** |

| **Contralateral** |  |  |
| --- | --- | --- |
|  |  |  |
|  | **PUMA** | **b-Actin** |
| **Sham-C** | 20.08 | 16.92 |
|  | 19.65 | 16.53 |
|  | 18.87 | 16.24 |
|  | 20.25 | 17.40 |
|  | 19.29 | 16.45 |
|  | 20.24 | 17.48 |
| **Average** | **19.73** | **16.84** |
| **SD** | **0.56** | **1.10** |
|  |  |  |
| **tIRI-C** | 19.99 | 16.63 |
|  | 19.29 | 16.59 |
|  | 19.35 | 16.32 |
|  | 19.06 | 16.27 |
|  | 19.15 | 16.43 |
|  | 19.39 | 16.47 |
| **Average** | **19.37** | **16.45** |
| **SD** | **0.33** | **0.77** |
|  |  |  |
| **FBP-C** | 19.20 | 16.63 |
|  | 19.13 | 16.43 |
|  | 20.14 | 17.47 |
|  | 20.09 | 16.65 |
|  | 19.62 | 16.44 |
|  | 20.21 | 17.09 |
| **Average** | **19.73** | **16.79** |
| **SD** | **0.49** | **0.41** |
|  |  |  |
|  |  |  |
|  |  |  |
|  |  |  |
|  |  |  |
|  |  |  |
| **Ipsilateral** |  |  |
|  |  |  |
|  | **PUMA** | **b-Actin** |
| **Sham-I** | 19.28 | 16.59 |
|  | 20.16 | 17.47 |
|  | 19.11 | 16.39 |
|  | 20.19 | 17.26 |
|  | 19.40 | 16.58 |
|  | 19.50 | 16.74 |
| **Average** | **19.61** | **16.84** |
| **SD** | **0.46** | **0.43** |
|  |  |  |
| **tIRI-I** | 18.04 | 16.47 |
|  | 18.94 | 16.36 |
|  | 19.35 | 17.07 |
|  | 18.28 | 16.19 |
|  | 18.35 | 16.58 |
|  | 18.07 | 16.40 |
| **Average** | **18.50** | **16.51** |
| **SD** | **0.52** | **0.30** |
|  |  |  |
| **FBP-I** | 20.81 | 17.48 |
|  | 19.85 | 16.81 |
|  | 18.91 | 16.33 |
|  | 19.60 | 17.26 |
|  | 18.78 | 16.40 |
|  | 19.04 | 16.93 |
| **Average** | **19.50** | **16.87** |
| **SD** | **0.77** | **0.46** |

| **Contralateral** |  |  |
| --- | --- | --- |
|  |  |  |
|  | **Survivin** | **b-Actin** |
| **Sham-C** | 22.42 | 16.916 |
|  | 22.306 | 16.533 |
|  | 21.545 | 16.239 |
|  | 22.436 | 17.4 |
|  | 21.72 | 16.453 |
|  | 23.102 | 17.482 |
| **Average** | **22.25** | **16.84** |
| **SD** | **0.56** | **1.10** |
|  |  |  |
| **tIRI-C** | 22.519 | 16.628 |
|  | 22.249 | 16.593 |
|  | 21.784 | 16.318 |
|  | 21.676 | 16.27 |
|  | 21.348 | 16.43 |
|  | 21.501 | 16.47 |
| **Average** | **21.85** | **16.45** |
| **SD** | **0.45** | **0.77** |
|  |  |  |
| **FBP-C** | 22.032 | 16.634 |
|  | 22.11 | 16.432 |
|  | 22.941 | 17.47 |
|  | 21.751 | 16.653 |
|  | 21.967 | 16.435 |
|  | 22.564 | 17.089 |
| **Average** | **22.23** | **16.79** |
| **SD** | **0.44** | **0.41** |
|  |  |  |
|  |  |  |
|  |  |  |
|  |  |  |
|  |  |  |
|  |  |  |
| **Ipsilateral** |  |  |
|  |  |  |
|  | **Survivin** | **b-Actin** |
| **Sham-I** | 21.162 | 16.588 |
|  | 22.205 | 17.471 |
|  | 21.663 | 16.386 |
|  | 22.356 | 17.256 |
|  | 21.284 | 16.575 |
|  | 21.318 | 16.735 |
| **Average** | **21.66** | **16.84** |
| **SD** | **0.51** | **0.43** |
|  |  |  |
| **tIRI-I** | 21.966 | 16.465 |
|  | 21.956 | 16.364 |
|  | 22.295 | 17.073 |
|  | 21.671 | 16.19 |
|  | 22.02 | 16.58 |
|  | 21.704 | 16.40 |
| **Average** | **21.94** | **16.51** |
| **SD** | **0.23** | **0.30** |
|  |  |  |
| **FBP-I** | 22.77 | 17.484 |
|  | 21.57 | 16.809 |
|  | 20.86 | 16.331 |
|  | 21.722 | 17.259 |
|  | 21.15 | 16.404 |
|  | 21.4 | 16.927 |
| **Average** | **21.58** | **16.87** |
| **SD** | **0.66** | **0.46** |
