# Supplementary material for: Altered expression profile of glycolytic enzymes during testicular ischemia reperfusion injury is associated with the p53/TIGAR pathway: effect of fructose 1,6-diphosphate
Source: PeerJ. 2016 Jul 5;4:e2195. doi: 10.7717/peerj.2195 (PMC4941766; doi:10.7717/peerj.2195)
Supplement: Data S2 [file peerj-04-2195-s002.docx]

**Glycolytic Enzymes Activities**

**HK1S**

| Sham - I | tIRI - I | FBP - I | Sham - C | tIRI - C | FBP - C |
| --- | --- | --- | --- | --- | --- |
| 2.195531 | 1.565906 | 1.593084 | 1.211542 | 2.170034 | 2.422782 |
| 2.422631 | 1.295153 | 2.305011 | 1.581469 | 2.00261 | 1.921673 |
| 1.800093 | 0.923333 | 1.616884 | 1.500929 | 1.264995 | 1.394485 |
| 1.726737 | 1.071306 | 2.147076 | 1.822181 | 1.104678 | 0.590022 |
| 1.924968 | 0.824253 | 1.403279 | 1.701365 | 1.500665 | 0.930372 |
| 2.392767 | 0.979801 | 2.168543 | 2.485117 | 2.233567 | 2.053751 |

No outliers identified by Grubbs test and/or Rout test.

**PGI**

| Sham - I | tIRI - I | FBP - I | Sham - C | tIRI - C | FBP - C |
| --- | --- | --- | --- | --- | --- |
| 0.996045 | 0.958706 | 0.484066 | 0.884017 | 0.685068 | 0.753906 |
| 0.796993 | 0.774535 | 0.932226 | 0.609547 | 0.837439 | 0.634831 |
| 0.997735 | 0.666223 | 0.613433 | 0.647926 | 0.921637 | 0.850486 |
| 0.736613 | 0.626065 | 0.687076 | 0.709816 | 0.927235 | 0.805917 |
| 0.952905 | 0.781124 | 0.836377 | 0.917777 | 0.916847 | 0.894987 |
| 0.986413 | 0.813455 | 0.702356 | 0.605844 | 0.974382 | 0.813056 |

No outliers identified by Grubbs test and/or Rout test.

**PFK**

| Sham - I | tIRI - I | FBP - I | Sham - C | tIRI - C | FBP - C |
| --- | --- | --- | --- | --- | --- |
| 1.184811 | 0.71816 | 1.247773 | 1.250684 | 1.226268 | 1.410329 |
| 1.129706 | ~~1.33463~~ | 1.278872 | 1.434707 | 1.566392 | 1.469761 |
| 1.225972 | 0.91924 | 1.048277 | 1.121397 | 0.925082 | 1.367168 |
| 1.265928 | 0.86329 | 1.522943 | 0.999678 | 1.156876 | 1.771192 |
| 1.405921 | 0.74903 | 1.238619 | 1.580491 | 0.754187 | 1.514861 |
| 1.790373 | 0.67768 | 1.484356 | 1.202351 | 0.84284 | 0.988855 |

~~123~~ Outliers identified by Grubbs test and/or Rout.

Re-analyzed data

| Sham - I vs. tIRI - I | Yes | ** | 0.0016 |
| --- | --- | --- | --- |
| tIRI - I vs. FDP - I | Yes | ** | 0.0029 |
| Sham - C vs. tIRI - C | No | ns | 0.5134 |
| tIRI - C vs. FDP - C | No | ns | 0.0549 |

**GAPDHS**

| Sham - I | tIRI - I | FBP - I | Sham - C | tIRI - C | FBP - C |
| --- | --- | --- | --- | --- | --- |
| 1.648403 | 1.218787 | 1.230013 | 1.232875 | 1.218787 | 1.230013 |
| 1.601458 | 1.258908 | 1.577287 | 1.1703 | 1.508908 | 1.577287 |
| 1.300598 | 0.818684 | 1.282706 | 1.52035 | 1.318684 | 1.282706 |
| 1.610642 | 1.050235 | 1.545187 | 1.5646 | 1.050235 | 1.545187 |
| 1.377058 | 1.187272 | 1.916763 | 1.3571 | 1.293858 | 1.416763 |
| 1.431265 | 0.759418 | 1.192881 | 1.33985 | 1.519551 | 1.192881 |

No outliers identified by Grubbs test and/or Rout test.

**PGK**

| Sham - I | tIRI - I | FBP - I | Sham - C | tIRI - C | FBP - C |
| --- | --- | --- | --- | --- | --- |
| 1.072235 | 0.654697 | 0.829358 | 0.995177 | 0.843866 | 1.042583 |
| 1.034665 | 0.906057 | 1.058512 | 1.026725 | 0.688871 | 0.679495 |
| 0.994825 | 0.738436 | 0.720684 | 1.076464 | 1.073619 | 0.972655 |
| 0.716753 | 0.793048 | 0.657965 | 0.781883 | 0.729156 | 0.602738 |
| 0.758749 | 0.688705 | 0.728991 | 0.58573 | 0.962369 | 0.748092 |
| 1.286048 | 0.819889 | 0.846591 | 1.073942 | 0.727282 | 0.909203 |

No outliers identified by Grubbs test and/or Rout test.

**LDHC**

| Sham - I | tIRI - I | FBP - I | Sham - C | tIRI - C | FBP - C |
| --- | --- | --- | --- | --- | --- |
| 1.639532 | 1.116139 | 1.735277 | 1.898403 | 1.968787 | 1.480013 |
| 2.475881 | 1.150883 | 2.087633 | 2.101458 | 2.258908 | 1.827287 |
| 1.896375 | 1.319764 | 1.563052 | 2.475598 | 2.068684 | 2.282706 |
| 2.102192 | 0.809436 | 1.879045 | 2.110642 | 1.780024 | 1.545187 |
| 2.481133 | 1.066979 | 1.595334 | 2.127058 | 2.114874 | 2.441676 |
| 1.509117 | 0.975765 | 1.801287 | 1.431265 | 1.972351 | 1.442881 |

No outliers identified by Grubbs test and/or Rout test.
