# Supplementary material for: Altered expression profile of glycolytic enzymes during testicular ischemia reperfusion injury is associated with the p53/TIGAR pathway: effect of fructose 1,6-diphosphate
Source: PeerJ. 2016 Jul 5;4:e2195. doi: 10.7717/peerj.2195 (PMC4941766; doi:10.7717/peerj.2195)
Supplement: Data S3 [file peerj-04-2195-s003.docx]

Histological data

**TUNEL**

| Sham - I | tIRI - I | FDP - I | Sham - C | tIRI - C | FDP - C |
| --- | --- | --- | --- | --- | --- |
| 1 | 44 | 5 | 1 | 19 | 5 |
| 0 | 36 | 9 | 0 | 15 | 8 |
| 0 | 38 | 16 | 0 | 18 | 12 |
| 1 | 24 | 6 | 0 | 17 | 8 |
| 2 | 39 | 8 | 1 | 22 | 14 |
| 1 | 51 | 15 | 0 | 24 | 6 |

No outliers identified by Grubbs test and/or Rout test.

**TBS**

| Sham - I | tIRI - I | FDP - I | Sham - C | tIRI - C | FDP - C |
| --- | --- | --- | --- | --- | --- |
| 10 | 6 | 7 | 10 | 8 | 9 |
| 10 | 6 | 8 | 10 | 8 | 9 |
| 8 | 5 | 8 | 9 | 9 | 10 |
| 10 | 6 | 8 | 10 | 10 | 8 |
| 10 | 6 | 7 | 10 | 9 | 8 |
| 8 | 5 | 8 | 9 | 10 | 9 |

No outliers identified by Grubbs test and/or Rout test.

**P53 Phosphorylation**

| Sham - T | Sham - Ph | tIRI - T | tIRI - Ph | FBP - Ph | FBP - T | Sham - T -C | Sham - Ph -C | tIRI - T -C | tIRI - Ph -C | FBP - Ph -C | FBP - T -C |
| --- | --- | --- | --- | --- | --- | --- | --- | --- | --- | --- | --- |
| 0.826291 | 1.004093 | 0.619632 | 1.660444 | 1.199036 | 1.361961 | 0.837722 | 0.717183 | 0.845249 | 0.850893 | 0.94769 | 0.820948 |
| 0.929915 | 0.864066 | 0.837623 | 1.74803 | 0.636082 | 0.753569 | 0.809591 | 0.812276 | 0.799884 | 0.715458 | 0.905331 | 0.727578 |
| 0.709105 | 1.034442 | 0.853404 | 1.497934 | 0.84668 | 0.80776 | 0.911231 | 0.693368 | 0.920106 | 0.921795 | 0.695183 | 0.923352 |
| 0.534032 | 0.865158 | 0.757063 | 1.610429 | 0.961095 | 1.09069 | 0.839726 | 0.839392 | 0.629643 | 0.734285 | 0.708805 | 0.915781 |
| 0.952433 | 0.900144 | 0.929589 | 1.157475 | 1.175797 | 0.985575 | 0.633938 | 0.931385 | 0.829767 | 0.617322 | 0.83976 | 0.817817 |
| 0.707727 | 0.740229 | 0.840667 | 1.175061 | 0.966606 | 0.966953 | 0.716938 | 0.831048 | 0.938334 | 0.827106 | 0.931612 | 0.915275 |

No outliers identified by Grubbs test and/or Rout test.

**P53 – IHC**

| Sham - I | tIRI - I | FBP - I | Sham - C | tIRI - C | FBP - C |
| --- | --- | --- | --- | --- | --- |
| 852 | 73500 | 10901 | 1095 | 820 | 1809 |
| 1112 | 56199 | 1365 | 2151 | 1346 | 1300 |
| 340 | 30162 | 5353 | 885 | 865 | 1219 |
| 1380 | 62684 | 2077 | 1074 | 1051 | 1068 |
| 1521 | 30344 | 36036 | 1095 | 1286 | 911 |
| 1346 | 38944 | 6136 | 950 | 958 | 1491 |
| 885 | 38013 | 3636 | 1249 | 1533 | 2029 |
| 979 | 44363 | 13636 | 2713 | 1447 | 737 |
| 1170 | 36403 | 7379 | 1727 | 1048 | 795 |
| 950 | 34497 | 4054 | 1298 | 2185 | 939 |

No outliers identified by Grubbs test and/or Rout test.

**TIGAR – IHC**

| Sham - I | tIRI - I | FBP - I | Sham - C | tIRI - C | FBP - C |
| --- | --- | --- | --- | --- | --- |
| 12801 | 265428 | 71791 | 60815 | 97268 | 74808 |
| 16192 | 253106 | 158085 | 39087 | 36454 | 63158 |
| 14006 | 250342 | 59639 | 39234 | 88923 | 82863 |
| 56552 | 217561 | 179855 | 51302 | 13480 | 83727 |
| 38272 | 231693 | 50995 | 34147 | 22080 | 25073 |
| 65975 | 346627 | 83480 | 54153 | 13354 | 32000 |
| 57951 | 130794 | 77491 | 13996 | 17179 | 54488 |
| 38007 | 174574 | 65874 | 15823 | 10339 | 74808 |
| 78591 | 206978 | 51590 | 19984 | 13936 | 26756 |
| 49087 | 155016 | 152439 | 21330 | 25377 | 24900 |

No outliers identified by Grubbs test and/or Rout test.
