# Supplementary material for: Altered expression profile of glycolytic enzymes during testicular ischemia reperfusion injury is associated with the p53/TIGAR pathway: effect of fructose 1,6-diphosphate
Source: PeerJ. 2016 Jul 5;4:e2195. doi: 10.7717/peerj.2195 (PMC4941766; doi:10.7717/peerj.2195)
Supplement: Data S4 [file peerj-04-2195-s004.docx]

**Biochemical Assays**

**SOD**

| Sham - I | tIRI - I | FDP - I | Sham - C | tIRI - C | FDP - C |
| --- | --- | --- | --- | --- | --- |
| 91.9297 | 82.3602 | 92.7962 | 96.4489 | 91.0684 | 87.5845 |
| 94.7340 | 81.9056 | 94.3078 | 96.4524 | 95.0829 | 96.2485 |
| 99.5445 | 81.2437 | 91.9601 | 94.5589 | 87.2943 | 89.4032 |
| 96.9732 | 79.9747 | 94.2430 | 91.0507 | 96.8901 | 78.8917 |
| 91.3846 | ~~75.6884~~ | 89.7895 | ~~78.1960~~ | 92.1638 | 91.4094 |
| 99.4649 | 80.6461 | ~~81.1477~~ | 92.0927 | 89.6912 | 99.6291 |

~~123~~ Outliers identified by Grubbs test and/or Rout test.

Re-analyzed data

| Sidak's multiple comparisons test | Significant? | Summary | Adjusted P Value |
| --- | --- | --- | --- |
|  |  |  |  |
| Sham - I vs. tIRI - I | Yes | **** | < 0.0001 |
| tIRI - I vs. FDP - I | Yes | *** | 0.0004 |
| Sham - C vs. tIRI - C | No | ns | 0.8660 |
| tIRI - C vs. FDP - C | No | ns | 0.9467 |

**CAT**

| Sham - I | tIRI - I | FDP - I | Sham - C | tIRI - C | FDP - C |
| --- | --- | --- | --- | --- | --- |
| 99.0380 | 67.6690 | 97.7472 | 97.2715 | 87.6501 | 97.2522 |
| 98.5157 | 84.9832 | 98.4411 | 93.8521 | 94.3097 | 98.2220 |
| 92.8446 | 82.0896 | 98.3063 | 90.5024 | 91.1321 | 96.9369 |
| 91.2038 | 81.5199 | 94.3748 | 87.8190 | 90.0946 | 96.9197 |
| 96.5268 | 85.2294 | 89.7741 | 96.6037 | 94.9166 | 97.8091 |
| 93.7682 | 93.8066 | 89.8490 | 98.3703 | 95.7910 | 95.3830 |

No outliers identified by Grubbs test and/or Rout test.

**GSH**

| Sham - I | tIRI - I | FDP - I | Sham - C | tIRI - C | FDP - C |
| --- | --- | --- | --- | --- | --- |
| 0.2714 | 0.3450 | 0.2319 | 0.2158 | 0.1851 | ~~0.2520~~ |
| 0.2290 | 0.3546 | 0.2288 | 0.2238 | 0.2167 | 0.1897 |
| 0.2545 | 0.2755 | ~~0.3031~~ | 0.2158 | 0.1953 | 0.1958 |
| 0.3190 | 0.3646 | 0.2347 | 0.2554 | 0.1907 | 0.1849 |
| 0.2416 | 0.2944 | 0.2241 | 0.2075 | 0.2266 | 0.1940 |
| 0.2154 | 0.3169 | 0.2529 | 0.1978 | 0.2256 | 0.1897 |

~~123~~ Outliers identified by Grubbs test and/or Rout test.

Re-analyzed data

| Sidak's multiple comparisons test | Significant? | Summary | Adjusted P Value |
| --- | --- | --- | --- |
|  |  |  |  |
| Sham - I vs. tIRI - I | Yes | *** | 0.0002 |
| tIRI - I vs. FDP - I | Yes | **** | < 0.0001 |
| Sham - C vs. tIRI - C | No | ns | 0.8562 |
| tIRI - C vs. FDP - C | No | ns | 0.7611 |

**MDA**

| Sham - I | tIRI - I | FDP - I | Sham - C | tIRI - C | FDP - C |
| --- | --- | --- | --- | --- | --- |
| 1.1600 | 1.9078 | 1.2079 | 1.0142 | 1.0263 | 0.9486 |
| 1.2741 | 1.4579 | 1.2978 | 1.0703 | 0.9124 | 0.9629 |
| 0.7531 | 1.7463 | 0.6923 | 1.0200 | 0.9583 | 1.0761 |
| 1.0351 | 1.8126 | 1.2459 | 1.1426 | 0.9304 | 1.1533 |
| 1.2129 | 1.0929 | 0.8301 | 0.9523 | 0.9760 | 0.9724 |
| 0.8933 | 2.0583 | 0.9652 | 0.9486 | 1.0850 | 0.9603 |

No outliers identified by Grubbs test and/or Rout.

**ATP**

| Sham-I | tIRI-I | FDP-I | Sham-C | tIRI-C | FDP-C |
| --- | --- | --- | --- | --- | --- |
| 0.5409 | 0.4787 | 0.6883 | 0.5055 | 0.6266 | 0.6945 |
| 0.5771 | 0.3422 | 0.7352 | 0.6814 | 0.5814 | 0.5534 |
| 0.5430 | 0.4905 | 0.4252 | 0.4542 | 0.5466 | 0.4494 |
| 0.6689 | 0.2999 | 0.5281 | 0.8294 | 0.5288 | 0.5052 |
| 0.6403 | 0.3432 | 0.5509 | 0.5084 | 0.4950 | 0.5710 |
| 0.5933 | 0.4833 | 0.4787 | 0.4950 | 0.6147 | 0.6375 |

No outliers identified by Grubbs test and/or Rout.

**NADPH**

| Sham-I | tIRI-I | FBP-I | Sham-C | tIRI-C | FBP-C |
| --- | --- | --- | --- | --- | --- |
| 2.7943 | 1.8284 | 2.7105 | 2.4036 | 1.2385 | 2.4073 |
| 2.5913 | 1.2914 | 2.5085 | 1.3403 | 2.4313 | 1.5594 |
| 3.0612 | 2.0480 | 2.5432 | 2.4315 | 1.3557 | 2.2497 |
| 2.7908 | 1.9605 | 1.9972 | 2.0204 | 2.3029 | 1.4537 |
| 3.3924 | 1.3042 | 2.4428 | 2.3860 | 2.4328 | 2.1018 |
| 2.6308 | 2.0579 | 2.9523 | 1.4506 | 1.4219 | 1.2366 |

No outliers identified by Grubbs test and/or Rout.
